# Unraveling the Core Components and Critical Targets of *Houttuynia cordata* Thunb. in Treating Non-small Cell Lung Cancer through Network Pharmacology and Multi-omics Analysis

**DOI:** 10.2174/0113816128330427241017110325

**Published:** 2024-10-21

**Authors:** Jinyan Yang, Yang Li, Yan Zhang, Ling Xu, Jiahui Wang, Feng Xing, Xinqiang Song

**Affiliations:** 1 College of Life Science, Xinyang Normal University, Xinyang 464000, China;; 2 Department of Ultrasound, Xinyang Central Hospital, Xinyang 464000, China;; 3 College of International Education, Xinyang Normal University, Xinyang 464000, China;; 4 Medical College, Xinyang Normal University, Xinyang 464000, China

**Keywords:** *Houttuynia cordata* Thunb., non-small cell lung cancer, network pharmacology, molecular docking, molecular dynamics simulation, drug targets

## Abstract

**Objective:**

This study aimed to preliminary explore the molecular mechanisms of *Houttuynia cordata* Thunb. (*H. cordata*; Saururaceae) in treating non-small cell lung cancer (NSCLC), with the goal of screening drug potential targets for clinical drug development.

**Methods:**

This study employed a multi-omics and multi-source data integration approach to identify potential therapeutic targets of *H. cordata* against NSCLC from the TCMSP database, GEO database, BioGPS database, Metascape database, and others. Meanwhile, target localization was performed, and its possible mechanisms of action were predicted. Furthermore, dynamics simulations and molecular docking were used for verification. Multi-omics analysis was used to confirm the selected key genes' efficacy in treating NSCLC.

**Results:**

A total of 31 potential therapeutic targets, 8 key genes, and 5 core components of *H. cordata* against NSCLC were screened out. These potential therapeutic targets played a therapeutic role mainly by regulating lipid and atherosclerosis, the TNF signaling pathway, the IL-17 signaling pathway, and others. Molecular docking indicated a stable combination between MMP9 and quercetin. Finally, through multi-omics analysis, it was found that the expression of some key genes was closely related not only to the progression and prognosis of NSCLC but also to the level of immune infiltration.

**Conclusion:**

Through comprehensive network pharmacology and multi-omics analysis, this study predicts that the core components of *H. cordata* play a role in treating NSCLC by regulating lipid and atherosclerosis, as well as the TNF signaling pathway. Among them, the anti-NSCLC activity of isoramanone is reported for the first time.

## 1 INTRODUCTION

Lung cancer is one of the malignant tumors with high morbidity and mortality worldwide, with NSCLC accounting for approximately 85% of all lung cancers [1], and lung adenocarcinoma (LUAD) and lung squamous cell carcinoma (LUSC) being the most common pathologic types [2]. With the emergence of various methods such as surgical treatment, radiotherapy, chemotherapy, targeted drug precise intervention, and cellular immunotherapy, modern medicine has made significant progress in the treatment of NSCLC. However, the prognosis of patients is still not satisfactory. This is due to several factors, including their inherent or acquired resistance to radiotherapy and chemotherapy, individual variations among patients, interference from coexisting underlying diseases, and the impact of genetic predispositions. Consequently, patients continue to encounter challenges such as elevated recurrence rates, substantial mortality rates, and significant metastasis rates. Among them, distant metastasis is an important cause of poor prognosis and high mortality in NSCLC patients [3]. According to incomplete statistics, 47.3% of NSCLC patients present with distant metastasis at diagnosis. The most frequent sites for metastasis include the brain, pleural, bone, liver, adrenal gland, and lung, with bone and lung metastasis being the most common [4, 5]. Therefore, for this type of metastatic tumor with genetic effects and cellular heterogeneity, it is of great significance to search for new targeted drugs for NSCLC patients with different subtypes and adopt personalized and precise treatment. As a traditional Chinese therapeutic measure, Chinese herbs have received increasing attention due to their multiple target pathways of action, weak drug resistance, and low side effects. Evidence from previous studies suggests that Chinese herbs have significant effects in preventing tumorigenesis, reducing tumor recurrence and metastasis, attenuating the toxicity of radiotherapy, enhancing the sensitivity of radiotherapy, and enhancing the immune function of the body [6-8].

*Houttuynia cordata* Thunb. (*H. cordata*) is a commonly used Chinese herbal medicine derived from the fresh whole plant or the dried aerial parts of the Saururaceae family. According to traditional Chinese medicine theory, *H. cordata* possesses a slightly cool and pungent flavor. Its therapeutic effects encompass clearing heat and detoxifying, eliminating stagnation and dispersing accumulations, relieving coughs, and dissipating phlegm. Primarily, it acts on the lung, liver, and heart meridians. Ancient Chinese medical texts often mention the use of *H. cordata* in the treatment of lung cancer. Modern medical research has demonstrated that *H. cordata* contains a variety of active ingredients [9-11], which can effectively alleviate inflammation and oxidative stress, induce apoptosis in NSCLC cells, inhibit their migration and proliferation, and exert numerous other functions, including immune regulation [8, 12-14]. However, further comprehensive research is still warranted to fully elucidate the mechanisms and material basis of *H. cordata* in the treatment of NSCLC.

In recent years, with the rapid development of bioinformatics research, especially the wide application of high-throughput technology, proteomics, genomics, transcriptomics, and other multi-omics, biology as a basic discipline has been greatly expanded. In this context, network pharmacology, a new discipline, came into being. With the help of network pharmacology, we can combine the targets of drugs and disease targets, predict the synergistic interactions between bioactive compounds [15], and study mechanisms of drug action from the overall perspective of biological networks, which is consistent with the overall concept and dialectical treatment theory of traditional Chinese medicine in treating diseases [16]. Utilized extensively in drug development, molecular docking is a sophisticated computer-based method [17]. By anticipating the interaction between ligands and targets at the molecular level or illustrating the relationship between structure-activity relationships, it can efficiently uncover novel compounds with therapeutic potential. Using network pharmacology and molecular docking techniques, Zhu *et al*. [18] predicted that *H. cordata* may alleviate pulmonary fibrosis through a variety of signaling pathways, including the PI3K/Akt pathways, MAPK pathways, TNF pathways, and IL-17 signaling pathways. Lai *et al*. [19] found that *H. cordata* has potential pharmacological effects on radiation-induced lung injury through the cancer pathways, TNF signaling pathway, and PI3K-Akt signaling pathway.

Molecular dynamics (MD) simulation, as an important tool for biomolecular computing research, is of great significance for analyzing the complex dynamic processes that occur in the studied biological systems by simulating the motion trajectories and states of molecular systems in the human environment using computers [20]. Based on comprehensive molecular dynamics simulations, Yuan *et al*. [21] confirmed that the various components of *H. cordata*, especially quercetin and kaempferol, may target multiple proteins to treat coronavirus disease 2019 (COVID-19) infection and COVID-19-induced cytokine storm. Gnanaselvan *et al*. [22] also screened 3',4',5,7-Tetrahydroxyisoflavanone (THIF), which may lead to the development of a potent anti-breast cancer through molecular docking, pharmacokinetics, and molecular dynamics simulation of wormwood. In summary, the research on network pharmacology and the application of molecular docking and dynamics simulation technology provide new insights into how traditional Chinese medicine interacts with diseases and drug development.

Additionally, gene chip data has been widely employed for drug screening and disease diagnosis due to the ongoing research on the human genome [23, 24]. Through in-depth bioinformatics analysis of DNA microarray data, Chong *et al*. [25] successfully identified five important genes associated with the occurrence and progression of gastric cancer, offering novel insights for its early detection and treatment. Meanwhile, the research of Shi *et al*. [26] also showed that a novel approach to precisely controlling the molecular mechanisms of important molecules at various levels during pharmacological intervention can be obtained by fusing network pharmacology with multi-omics analysis. Furthermore, Zhang *et al*. [27] comprehensively utilized network pharmacology analysis, differential expression analysis of key target genes, and prognostic analysis in their study to unveil the potential mechanism of Aidi injection in the treatment of NSCLC, providing a scientific basis for further research on its treatment of NSCLC. Similarly, the important role of *H. cordata* in targeting the core targets of respiratory syncytial virus has been validated through bioinformatics data mining [28]. Therefore, by combining the systematic thinking of network pharmacology with comprehensive data from bioinformatics, we can gain a deeper understanding of the interaction between drugs and biological systems, thereby accelerating the discovery and development of new drugs. This comprehensive research method will greatly impact the field of drug development and provide powerful tools for future pharmaceutical innovation.

In this study, we utilized network pharmacology, molecular docking, molecular dynamics simulation, and other bioinformatics techniques to analyze the multi-component target action network of *H. cordata* in treating NSCLC and to explore its underlying mechanisms of action at the molecular level. All these provide a theoretical basis for the follow-up study of *H. cordata* in the treatment of NSCLC and may provide valuable clues for finding new treatment opportunities. This not only helps to understand the mechanisms of action between drugs and biological systems but also provides important theoretical support for new drug research and development.

## 2 MATERIALS AND METHODS

In this study, the key active ingredients and action targets of *H. cordata* were mined from the aspects of target localization, enrichment analysis, computational biology, and multi-omics validation. The specific experimental process is shown in Fig. (**S1**).

### 2.1 Collection of Active Components of *H. cordata*

Using “Yuxingcao” as the keyword, the TCMSP (https://old.tcmsp-e.com/tcmsp.php) database was searched to screen all of the active components of *H. cordata* [29]. The selection of oral bioavailability (OB) ≥ 30% and drug-likeness (DL) ≥ 0.18 as screening criteria was based on the ADME system evaluation model [30]. At the same time, combined with the previous reports, considering the content, biological effects, and other factors, the active components of *H. cordata* to be studied were finally determined.

### 2.2 Prediction of Potential Targets of Active Components in *H. cordata*

The “Canonical SMILES” and “2D Structure” of the active components to be studied were obtained from PubChem (https://pubchem.ncbi.nlm.nih.gov) [31]. Then, to predict the potential targets of *H. cordata*, we imported them into the Swiss Target Prediction database (http://www.swisstargetprediction.ch/) [32], set the species to “*Homo sapiens*”, screened out targets with “Probability > 0”, and finally deduplicated the obtained targets.

### 2.3 Collection of Therapeutic Targets Associated with NSCLC

Using the keyword “non-small cell lung cancer” with the organism set to “*Homo sapiens*” and a sample size greater than 20, the GEO database (https://www.ncbi.nlm.nih.gov/geo) [33] was queried to gather gene chips associated with NSCLC. Subsequently, the online tool GEO2R (https://www.ncbi.nlm.nih.gov/geo/geo2r) was utilized to screen for differentially expressed genes between NSCLC and normal tissues [34], with filtering conditions set to “adj. *P*-value < 0.05” and “| log2 (foldchange)| ≥ 2”. Then, the differentially expressed genes were screened for the second time through the Bioinformatics&EvolutionaryGenomics online website (http://bioinformatics.psb.ugent.be/webtools/Venn), and the genes that were differentially expressed in at least two chips were retained.

### 2.4 Prediction of Potential Therapeutic Targets of *H. cordata* against NSCLC

The intersection of the potential targets of *H. cordata* obtained in “2.2” and the NSCLC-related targets obtained in “2.3” were the potential therapeutic targets of *H. cordata* against NSCLC.

### 2.5 Organ-tissue Localization Analysis of Potential Therapeutic Targets

In order to explore the potential relationship between *H. cordata* and NSCLC at the organ and tissue level, the BioGPS database (http://biogps.org/) was searched to obtain the mRNA expression data of the above potential therapeutic targets in different tissues [35], including the whole brain, thymus, bone marrow, liver, adrenal gland, lung, and so on. Then, the target tissue distribution was determined based on three times the median value of mRNA expression in different tissue types, and targets lacking available probes were excluded from this analysis [36]. Finally, the organ-tissue location network of potential therapeutic targets was constructed by Cytoscape 3.9.1 software.

### 2.6 Determination of Key Targets of *H. cordata* against NSCLC

The information about the potential therapeutic targets mentioned above was imported to the STRING database (https://cn.string-db.org/) [37], species were set as “*Homo sapiens*”, confidence as “0.4”, and hide free nodes to obtain the interaction relationships between proteins. After that, Cytoscape 3.9.1 software was used to plot the protein interaction diagram of these targets [38], and the CytoNCA plug-in was used to analyze network topology parameters. Nodes with a “Degree” greater than the average value were selected to obtain the key targets of *H. cordata* against NSCLC.

### 2.7 Enrichment Analysis of Potential Therapeutic Targets and Determination of Core Components

The potential therapeutic targets were entered into the Metascape database (http://metascape.org) [39] for Gene Ontology (GO) and Kyoto Encyclopedia of Genes and Genomes (KEGG) enrichment analysis. “Min Overlap” was set to 3, “*P*-value Cutoff” was set to 0.05, “Min Enrichment” was set to 1.5, and the species selected was “*H. sapiens*”. Then, through the SRplot platform (https://www.bioinformatics.com.cn/SRplot) [40], enrichment results were visualized. Meanwhile, the “disease-pathways-components-targets” network was constructed by integrating the relevant information about components, targets, and pathways. The network was topologically analyzed to obtain the core components of *H. cordata* against NSCLC. In addition, the pathways with better results were visualized based on the KEGG database (https://www.kegg.jp/) [41].

### 2.8 Molecular Docking and Molecular Dynamics Simulation of Core Components and Key Genes

To further study interactions between the core components and key genes, this study first conducted molecular docking of core components and key genes. The 3D structures of the core components and key genes were respectively obtained from the PubChem database and the RCSB PDB database (https://www.rcsb.org/) [42]. AutoDock Tools 1.5.6 was used to perform molecular docking of the key proteins and core ingredients, and the best conformation was drawn by PyMOL software. In addition, on the basis of molecular docking, molecular dynamics simulations were performed using GROMACS 2018.8 to assess the binding affinity and stability of the optimal conformation [43].

Origin software was utilized to analyze various parameters of the MD simulation, including root mean square deviation (RMSD), root mean square fluctuation (RMSF), radius of gyration (Rg), solvent-accessible surface area (SASA), and hydrogen bonds (Hb). These values, as important observations in the dynamic modeling process, can effectively measure the stability of the system during the simulation and reflect both the deformation of local points and the tightness of the system structure.

### 2.9 mRNA Expression Level Analysis, Survival Prognosis Analysis, and Immunohistochemical Analysis of Key Genes

Using the “Single Gene Analysis” module of GEPIA (http://gepia.cancer-pku.cn/) [44], the mRNA expression levels of key genes in NSCLC tissues and normal lung tissues were compared, and their relationship with pathological staging was analyzed. Subsequently, with the assistance of the Kaplan-Meier Plotter database (https://kmplot.com/analysis/) [45], the prognostic hazard ratio (HR), the risk ratio of the 95% confidence interval, and corresponding log-rank *P*-value were calculated, the corresponding survival curves were plotted, and the effects of these key genes' mRNA expression levels on the overall survival (OS) of NSCLC patients were compared to analyze the prognostic implications of each key gene for NSCLC patients. Based on this, co-expression analysis of genes with prognostic value was performed using the GEPIA platform. Finally, immunohistochemistry (IHC) staining images of key genes in lung cancer tissues and normal lung tissues were obtained from the HPA database (https://www.proteinatlas.org/) to conduct an in-depth study on the expression of key genes in NSCLC patients and healthy lung tissues at the protein level [46]. To ensure the accuracy and reliability of the study, we adhered to the following principles when selecting samples for this research: when studying the same gene, normal tissue images and pathological images with the same sex, similar age, and consistent antibody models for immunohistochemical detection were selected to reduce potential errors arising from differences in sex, age, and antibody models.

### 2.10 Analysis of Genetic Alterations of Key Genes in NSCLC Patients

The cBioPortal tool (https://www.cbioportal.org/) was utilized to cross-mine pertinent data for cancer genomics and examine changes in the genetic information of key genes in NSCLC tissues [47].

### 2.11 Analysis of Key Genes and Immune Infiltration in Tumor Microenvironment of NSCLC

By the TIMER database (https://cistrome.shinyapps.io/timer/) [48, 49], the expression of key genes and the correlation between somatic copy number variation and infiltration of six immune cells (B cells, CD4 + T cells, CD8 + T cells, neutrophils, macrophages, and dendritic cells) were analyzed. Then, in order to more fully understand the impact of this association on prognosis, this study also analyzed the relationship between the degree of infiltration of these immune cells in NSCLC tissues and the survival of patients. At the same time, the correlation between the expression of key genes and the markers of these immune cells was further explored.

## 3 RESULTS

### 3.1 Identification of Potential Therapeutic Targets of *H. cordata* against NSCLC

**Fig. (1):**
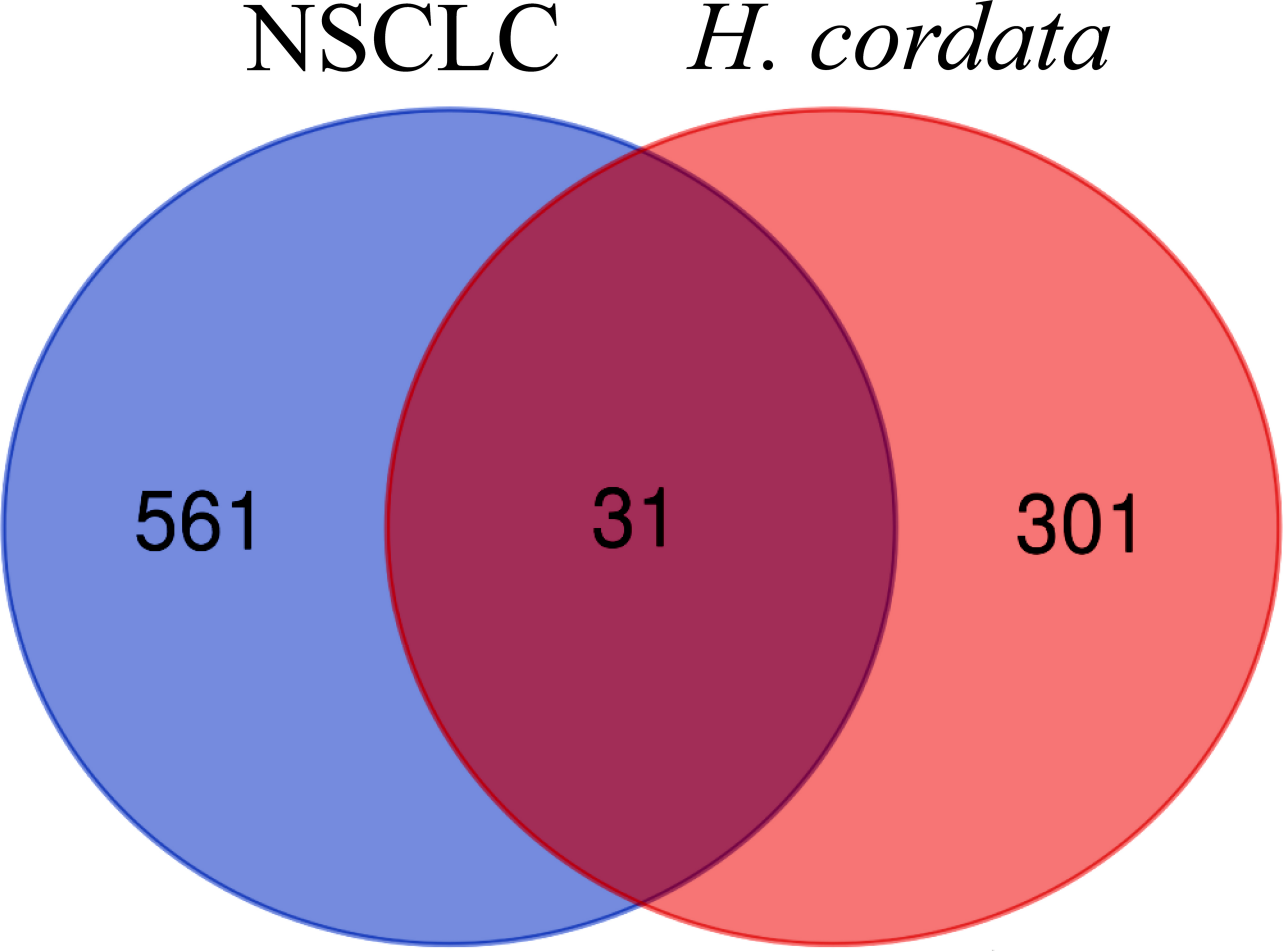
Venn diagram of the relationship between the potential targets of the active ingredients in *H. cordata* and the differentially expressed genes (DEGs) in NSCLC.

According to the TCMSP target screening guidelines, a total of seven potential active ingredients of *H. cordata* were identified in the TCMSP database: isoramanone, kaempferol, ruvoside_qt, spinasterol, quercetin, 1-methyl-2-nonacosyl-4-quinolone, and C09747. Previous pharmacological studies [9, 50, 51] indicated that the active components of *H. cordata* effective against NSCLC mainly include volatile oils, alkaloids, flavonoids, and polysaccharides. However, during the screening process in TCMSP, some of these components might have been filtered out. Therefore, we combined the relevant reports on the active components of *H. cordata* to supplement the missing active components and added 2-undecanone [10], decanal [52], decanoyl acetaldehyde (also known as houttuynin) [18], rutin [19], hyperin [21], quercitrin [52, 53], dodecanal (also known as lauric aldehyde) [54], and afzelin [52]. As shown in Tables **S1** and **S2**, we obtained 15 active ingredients for further investigation in total. Meanwhile, a total of 332 potential targets for the active components of *H. cordata* were predicted in the Swiss Target Prediction database. Among them, C09747 did not have any corresponding targets and was therefore excluded from subsequent analysis. In the GEO database, the expression profiling chip data utilized in this study were derived from the chips GSE19188, GSE19804, GSE18842, and GSE33532. Through differential expression analysis (Figs. **S2a**-**S2d**), a total of 592 differentially expressed genes were identified that were present in at least two different chips (Fig. **S2e**). Based on this, the 31 potential targets of the active ingredients in *H. cordata*, which were also among the 592 NSCLC-related differentially expressed genes, were identified (Fig. **1**).

### 3.2 Potential Association between *H. cordata* and NSCLC at the Organ-tissue Level

**Fig. (2):**
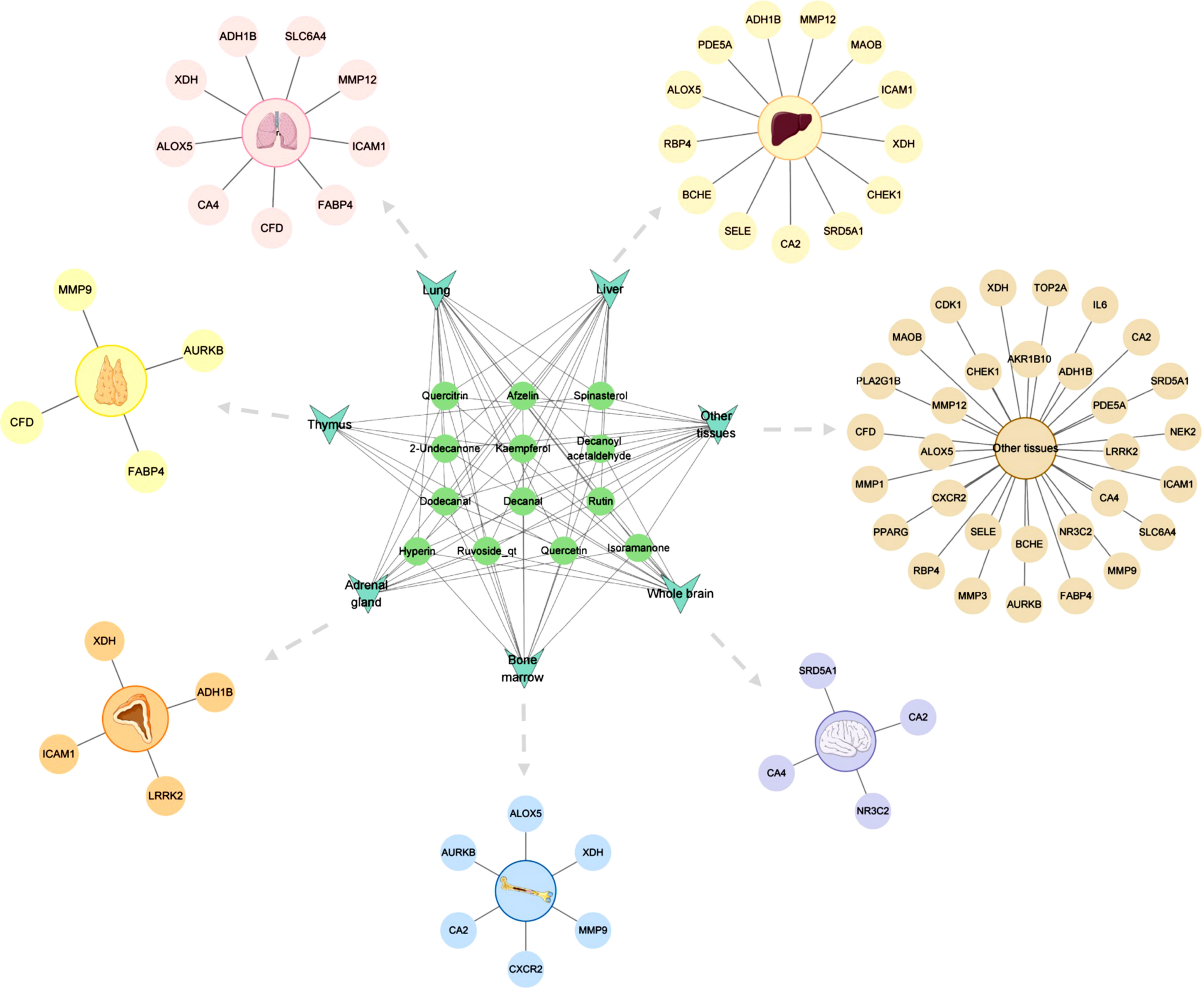
Organ-tissue localization network of potential therapeutic targets for *H. cordata* against NSCLC.

After constructing the organ-tissue location network of potential anti-NSCLC therapeutic targets of *H. cordata* (Fig. **2**), we found that a total of nine potential therapeutic targets were distributed in the lung, including ADH1B, XDH, ALOX5, CA4, CFD, FABP4, ICAM1, MMP12, and SLC6A4. This is consistent with the fact that the lung is the organ affected by NSCLC. Secondly, nine targets, including ADH1B, ALOX5, CA2, CA4, FABP4, ICAM1, MMP9, SRD5A1, and XDH, were found to be distributed in two or more sites: the whole brain, thymus, bone marrow, liver, and adrenal gland. This indicates that these tissues are closely related to each other in the development of NSCLC, and different components of *H. cordata* can act on different target sites in multiple tissues to exert the effect of treating NSCLC. At the same time, this is also consistent with the fact that these tissues are common sites of NSCLC metastasis.

### 3.3 Multiple Components in *H. cordata* Act on Multiple Targets to Regulate Multiple Pathways against NSCLC

**Fig. (3):**
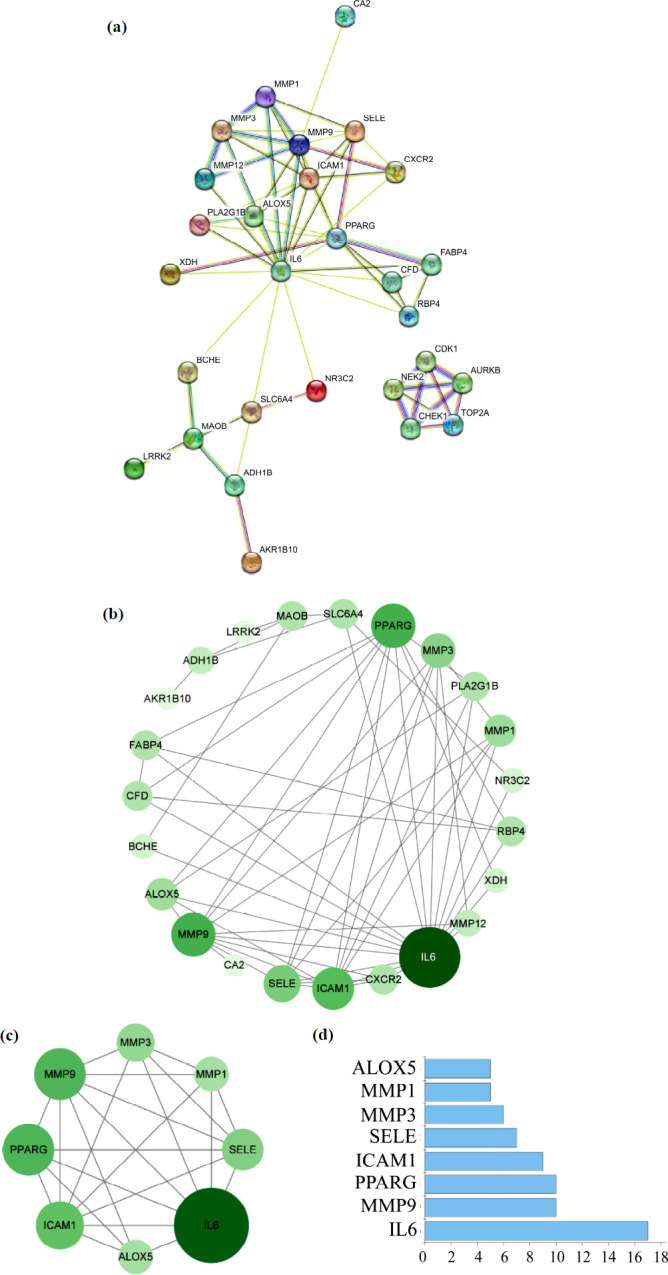
Screening of key targets of *H. cordata* against NSCLC. (**a** and **b**) PPI networks of potential therapeutic targets were constructed using the STRING database (**a**) and Cytoscape software (**b**). The greater the “Degree” value of each node, the darker the color and the larger the size. (**c**) Key targets were selected using Cytoscape software. (**d**) “Degree” values of key targets.

After importing the data on the aforementioned 31 potential therapeutic targets into the STRING database, we obtained interactions between the targeted proteins and constructed a preliminary protein-protein interaction (PPI) network diagram (Fig. **3a**). This network diagram comprised 31 nodes and 66 edges. Among these nodes, CA4, SRD5A1, and PDE5A showed no interactions with other proteins and were thus considered free nodes. Subsequently, we performed topological analysis on the remaining 28 targeted proteins and constructed a network of potential therapeutic targets (Fig. **3b**). Within this network, CDK1, NEK2, CHEK1, AURKB, and TOP2A formed an exclusive subnetwork that was not depicted in the diagram. Furthermore, we calculated the mean value of “Degree” for the entire network to be 4.870. Using the criterion of “Degree > 4.870”, we identified 8 key targets of *H. cordata* against NSCLC, as shown in Figs. (**3c** and **3d**): IL6, MMP9, PPARG, ICAM1, SELE, MMP3, MMP1, and ALOX5.

Next, GO analysis of the Metascape database yielded 338 GO entries, of which 294 entries were for biological processes (BP), cellular components (CC) covered 21 entries, and 23 molecular functions (MF). As illustrated in Fig. (**4a**), the primary biological processes involved in the anti-NSCLC effect of *H. cordata* included the collagen catabolism process, regulation of inflammatory response, regulation of hormone levels, and positive regulation of cell death. The cellular components were mostly enriched in regions such as the ficolin-1-rich granular lumen, mitotic spindle, and condensed chromosome. Additionally, the molecular functions were primarily related to histone kinase activity, metalloendopeptidase activity, serine-type endopeptidase activity, and serine hydrolase activity.

**Fig. (4):**
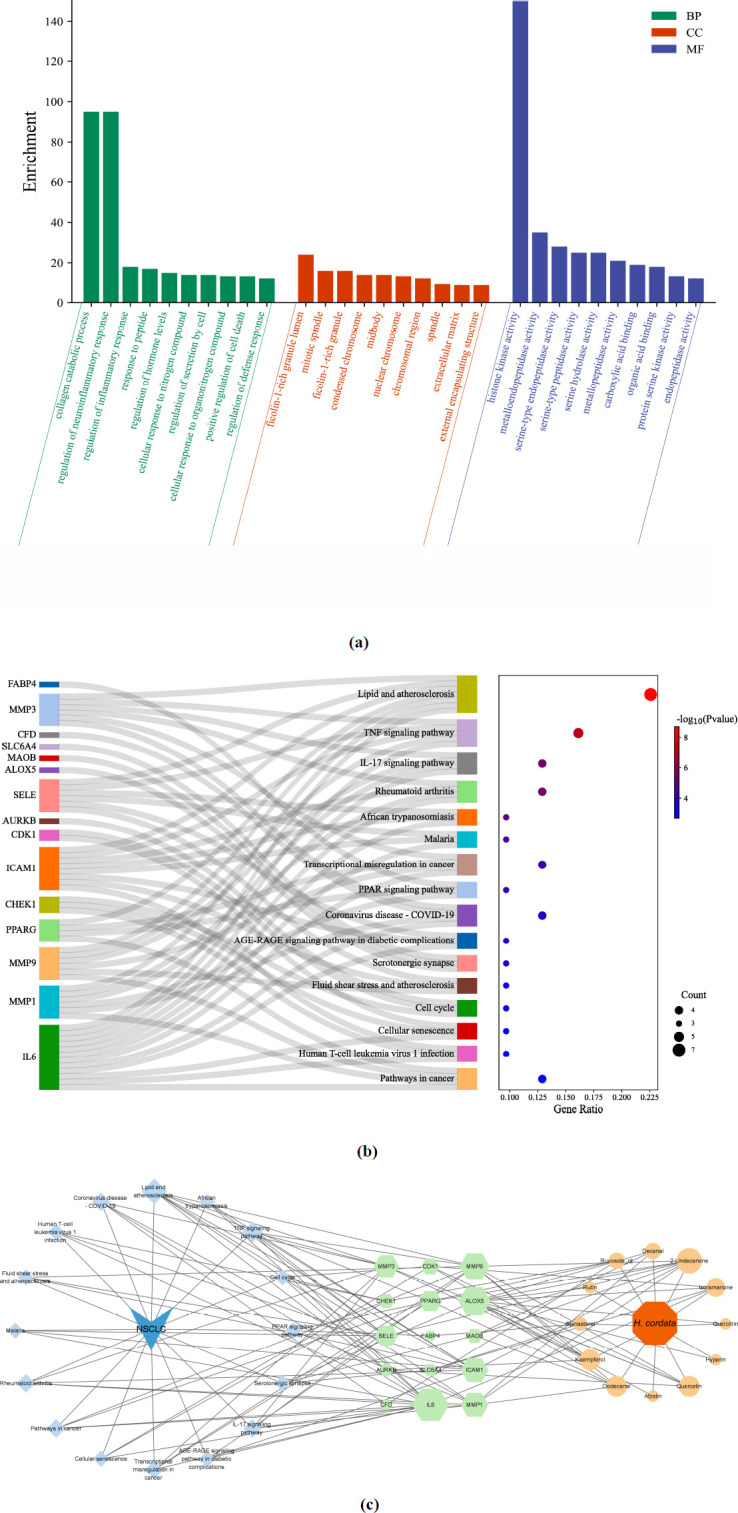
Identification of the potential function of *H. cordata*. (**a**) Top 10 BP terms, CC terms, and MF terms of GO enrichment analysis are shown as green, orange, and purple bars, respectively. (**b**) The Sankey diagram of the KEGG pathway analysis of the potential therapeutic targets of *H. cordata* in the treatment of NSCLC. The left rectangle nodes of the Sankey diagram represent the potential therapeutic targets, the right rectangle nodes of the Sankey diagram represent the KEGG pathways, and the lines represent the ownership of targets and pathways. The larger the bubble, the more potential therapeutic targets are enriched in this pathway. (**c**) The “disease-pathways-components-targets” network diagram. The green nodes represent the potential therapeutic targets, the light orange nodes represent the compounds, whereas the light blue nodes represent the pathways.

KEGG pathway enrichment analysis of potential therapeutic targets showed a total of 16 signaling pathways, the majority of which were associated with cancer (Fig. **4b**). Notably, the pathways that might be related to NSCLC included lipid and atherosclerosis, the TNF signaling pathway, the IL-17 signaling pathway, transcriptional misregulation in cancer, pathways in cancer, the PPAR signaling pathway, and others. Additionally, pathways associated with other diseases and immune responses included rheumatoid arthritis, African trypanosomiasis, COVID-19, human T-cell leukemia virus 1 infection, and so on. Notably, lipid and atherosclerosis, the TNF signaling pathway, rheumatoid arthritis, and the IL-17 signaling pathway were significantly enriched. These pathways serve crucial physiological functions in the organism and are intricately linked to the onset and progression of numerous diseases.

Based on the aforementioned data, we utilized Cytoscape 3.9.1 software to construct a “disease-pathways-components-targets” network (Fig. **4c**), which had 45 nodes and 125 edges, including 12 active components, 15 potential therapeutic targets, and 16 signaling pathways. Within this network, the size of the nodes is proportional to their “Degree” values; consequently, a larger node signifies a greater number of connections. This implies that distinct active components of *H. cordata* may regulate multiple pathways by acting on multiple targets to exert therapeutic effects on NSCLC, exemplifying the multi-component and multi-target nature of *H. cordata*. Among these components, 2-undecanone, dodecanal, kaempferol, quercetin, and isoramanone possess larger nodes, signifying their status as the core active components responsible for the anti-NSCLC effects of *H. cordata*. Simultaneously, based on topological analysis (Figs. **S3a**-**S3c**), it was observed that all eight key targets identified above exhibited high degrees of connectivity, highlighting their significance as vital targeted genes for the anti-NSCLC efficacy of *H. cordata* and as the linchpin for the interaction between its effective active components and the corresponding targets in the human body. Furthermore, both lipid and atherosclerosis and the TNF signaling pathway displayed notable degrees of connectivity (Figs. **4c** and **S3a**). Therefore, these two signaling pathways not only showed good differences but also enriched a large number of potential therapeutic targets (Figs. **S3d** and **S3e**), indicating their pivotal role in mediating the anti-NSCLC efficacy of *H. cordata*.

### 3.4 Core Components and Key Genes Exhibit Favorable Binding Energy as a General Trend

**Fig. (5):**
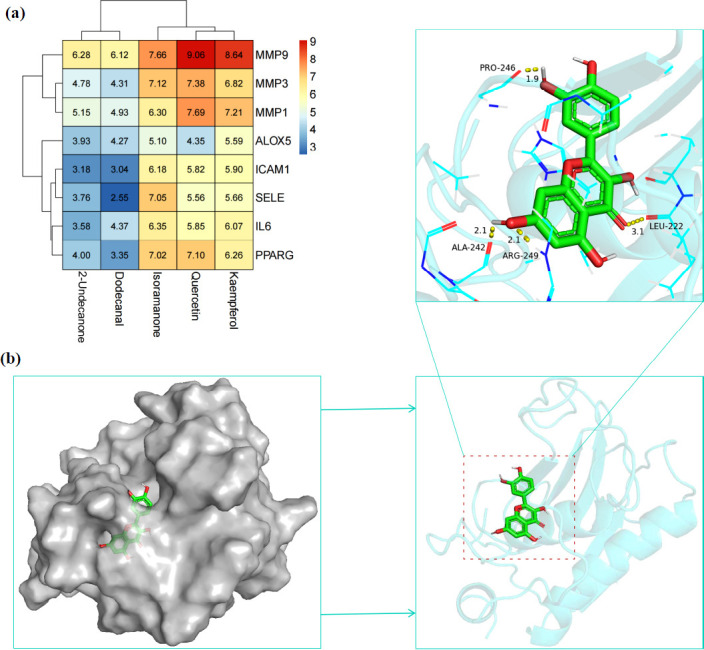
Molecular docking of key genes and core components. (**a**) Heat map of docking binding energy between core components and key genes. The redder the color, the smaller the binding energy, which means the stronger the binding ability. (**b**) Docking pattern of MMP9-Quercetin.

To better elucidate the degree of association and mode of action between each core component and the key gene, eight key genes were docked with five core components of *H. cordata*, and only the highest absolute value of binding energy for each group was retained in the docking results (Fig. **5a**). It is generally believed that the lower the binding energy, the more stable the binding between small molecules and receptor proteins, and the higher the affinity between the two. A binding energy <-4.25 kcal/mol indicates positive binding activity, an energy <-5.0 kcal/mol demonstrates suitable binding activity, and an energy <-7.0 kcal/mol indicates robust binding activity [55]. Hence, based on these docking results (Fig. **S4**), it can be observed that isoramanone exhibits good binding activity with MMP9, MMP3, SELE, and PPARG, while quercetin shows good binding affinity for MMP9, MMP3, MMP1, and PPARG. Additionally, kaempferol shows good binding with two key genes, MMP9 and MMP1. Notably, quercetin demonstrates the strongest binding activity with MMP9, completely entering its active pocket (Fig. **5b**) and forming hydrogen bonds with the active sites of MMP9, specifically LEU-222, ALA-242, PRO-246, and ARG-249. This suggests that the interaction between quercetin and MMP9 plays a pivotal role in the anti-NSCLC effect of *H. cordata*.

**Fig. (6):**
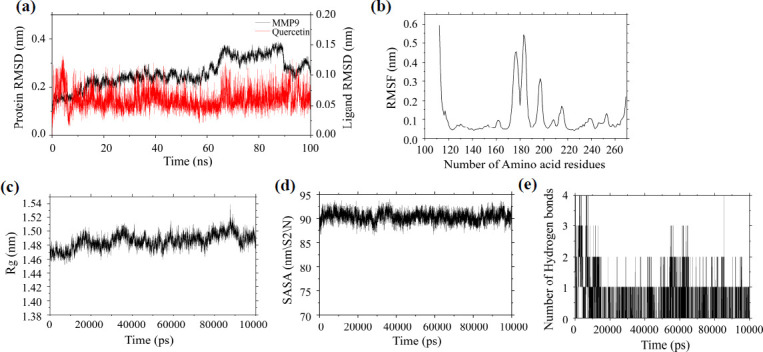
Molecular dynamics simulation of MMP9-Quercetin. (**a**) The RMSD of MMP9-Quercetin. (**b**) The RMSF of MMP9-Quercetin. (**c**) The Rg of MMP9-Quercetin. (**d**) The SASA of MMP9-Quercetin. (**e**) The Hb of MMP9-Quercetin.

To further validate the molecular docking results, this study conducted molecular dynamics simulations specifically on the most tightly bound combination of quercetin and MMP9. During the simulation, the RMSD of the MMP9 protein fluctuated slightly and tended to be flat after 20 ns (Fig. **6a**), and its average RMSD was less than 0.26 nm, indicating the good stability of the system. Analysis of RMSF (Fig. **6b**) revealed minimal overall conformational changes in the amino acids of the MMP9 protein, with an average RMSF of less than 0.12 nm. Using 0.12 as the fluctuation cutoff value and considering values greater than 0.12 as indicating higher volatility, it was observed that the system composed of quercetin and MMP9 exhibited high fluctuations in the 112-117, 173-186, 195-199, 213-216, 252-253, and 268-269 regions. Therefore, this MD simulation did cause a change in protein terminal conformation, but this change was probably caused by the loose packaging of its terminal, so the protein still had high stability in the simulation process. Subsequently, the Rg, SASA, and Hb were calculated (Figs. **6c**-**6e**). During the simulation, the Rg of the MMP9 protein remained consistently below 2 nm with minimal overall fluctuations. The SASA of the MMP9 protein remained stable throughout the simulation, although a slight downward trend was observed within the 20~30 ns range. Additionally, the MMP9-Quercetin complex maintained stable hydrogen bonds, with up to four hydrogen bonds observed at certain times and two or three hydrogen bonds present at other times.

In summary, the results of the MD simulation indicate that MMP9 and quercetin form a relatively stable molecular dynamics system that can stably exist without affecting their own or other protein structures, strongly supporting the validity of the docking results.

### 3.5 Significant Impact of mRNA Expression Levels of Key Genes on NSCLC Progression and Patient Prognosis, with Differential Protein Expression Profiles Observed in Normal *versus* NSCLC Tissues

**Fig. (7):**
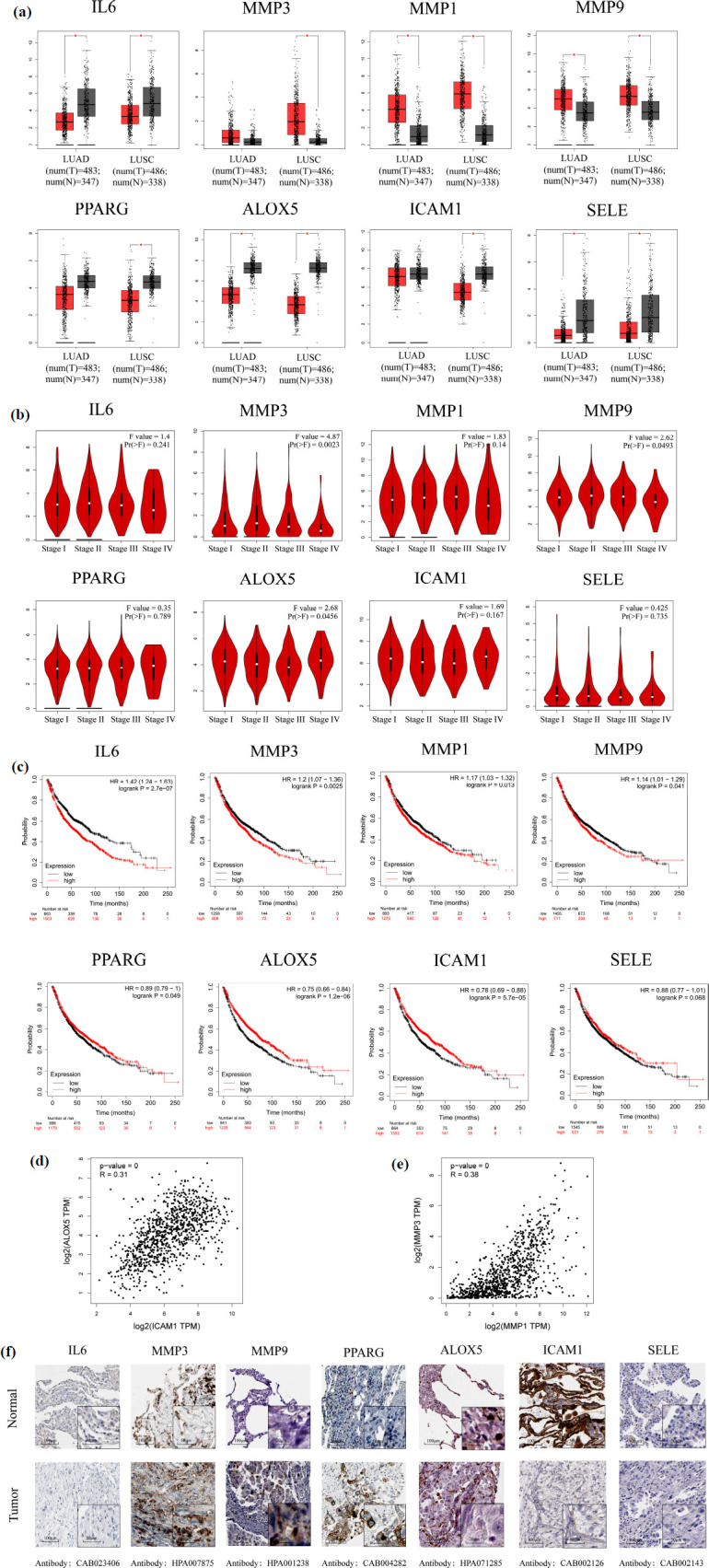
Analysis of the mRNA expression level, prognostic values, and protein expression level of key genes. (**a**) Boxplot of key genes mRNA expression levels in the GEPIA database. Red represents NSCLC tissue, and gray represents normal lung tissue. (**b**) Stage plot of key genes mRNA expression levels and pathological stages in the GEPIA database. (**c**) Kaplan-Meier overall survival analyses of patients with NSCLC based on expression of key genes. Grey represents low expression, and red represents high expression. (**d**) Analysis of the co-expression of ALOX5 and ICAM1. (**e**) Analysis of the co-expression of MMP3 and MMP1. (**f**) IHC images of key genes protein expression levels in the HPA database.

In the GEPIA database, a notable downregulation of IL6, PPARG, ICAM1, ALOX5, and SELE was observed in NSCLC tissues (*P* < 0.05), while MMP3, MMP9, and MMP1 demonstrated significant upregulation (Fig. **7a**). It was worth mentioning that MMP3, PPARG, and ICAM1 only displayed marked expressions in LUSC. Upon further examination of the correlation between the mRNA expression levels of these pivotal genes and the pathological staging of NSCLC, a considerable variation in the expression levels of MMP3, MMP9, and ALOX5 was evident across different pathological stages (Fig. **7b**). This finding hints at a potential association between the expression of these genes and the progression of NSCLC.

Subsequently, survival analysis was conducted using the Kaplan-Meier Plotter database. Apart from SELE, the expression of all other key genes was significantly correlated with the prognosis of NSCLC patients (Fig. **7c**). Specifically, the impacts of IL6, MMP3, ALOX5, and ICAM1 expression on the prognosis of NSCLC patients were highly significant (*P* < 0.01). More precisely, patients with high expression of ICAM1 and ALOX5 had significantly higher overall survival rates compared to those with low expression. Conversely, patients with low expression of IL6 and MMP3 had notably higher overall survival rates than those with high expression. Based on these findings, further analysis was performed to investigate the potential co-expression relationships among the seven prognostic genes. A significant positive correlation was observed between the expression of ALOX5 and ICAM1, as well as between MMP3 and MMP1 (Figs. **7d** and **7e**). This suggested potential interactions between ALOX5 and ICAM1, and, similarly between MMP3 and MMP1. However, the relationship between ALOX5 and ICAM1 has not been extensively explored in previous reports.

Finally, the IHC images in the HPA database showed that the other five genes were expressed in different degrees in normal lung tissues, except for IL6 and SELE (Fig. **7f**). Compared with normal lung tissues, the expression of MMP3, MMP9, and PPARG was increased in lung cancer tissues, and the expression of ALOX5 and ICAM1 was decreased in lung cancer tissues, which was basically consistent with the analysis results in the GEPIA database. However, the database lacked data related to MMP1, so the expression of MMP1 at the protein level was not analyzed.

### 3.6 Mutations in Key Genes Among NSCLC Patients

**Fig. (8):**
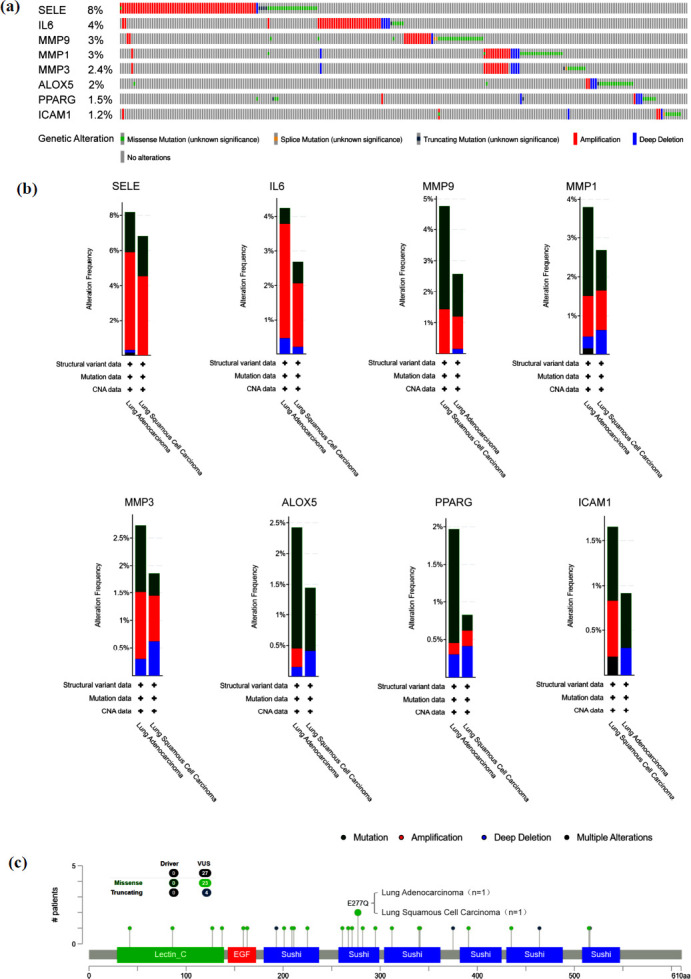
Analysis of genetic changes in key genes by cBioPortal. (**a**) Genetic change map of key genes. (**b**) Genomic changes of key genes. (**c**) A two-dimensional structure map of the protein corresponding to SELE.

Using the cBioPortal tool, we analyzed genetic data from NSCLC patients and found that alterations occurred in 287 out of 1144 patients (25.1%) for these key genes. These changes were primarily categorized as “Amplification” and “Mutation” (Figs. **8a** and **8b**). Notably, SELE exhibited an alteration frequency of 8%, and the main mutation type was “Missense Mutation” (Figs. **8a** and **8c**). Upon further analysis, it was revealed that the E277Q mutation within the SELE gene exhibited the highest correlation with overall SELE mutations, affecting one case of LUAD and one case of LUSC. This mutation, located in the Sushi domain of SELE, is suspected to have a significant impact on its functionality.

### 3.7 Effect of Key Gene Expression and Variation on Immune Infiltration Levels in NSCLC

**Fig. (9):**
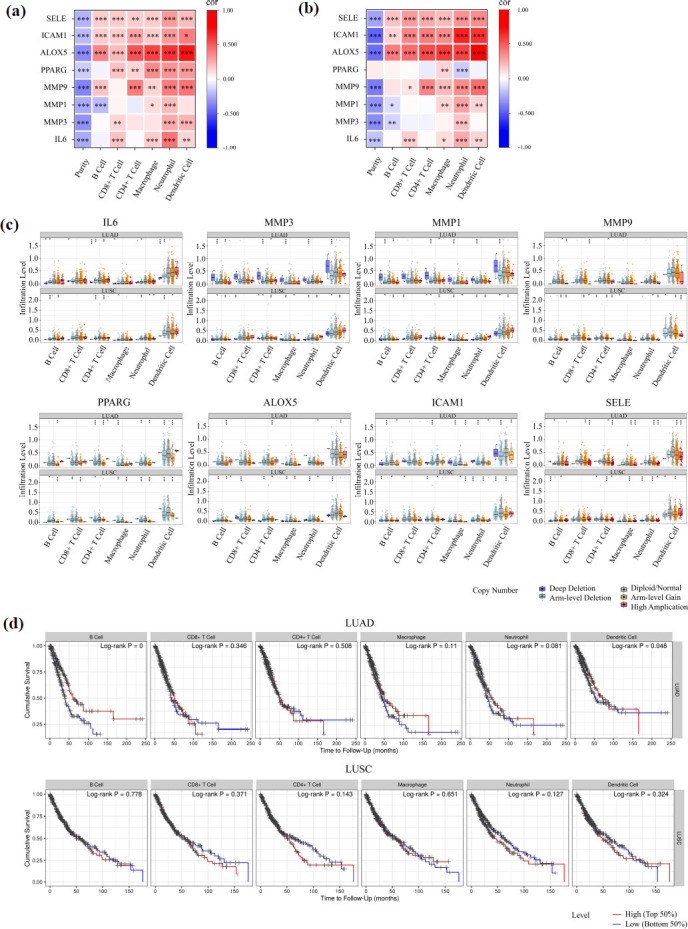
Correlation between the expression of key genes and immune infiltration. (**a**) Correlation between immune cell infiltration and expression levels of key genes in LUAD in the Timer database. Red indicates a positive correlation, and blue indicates a negative correlation. (**b**) Correlation between immune cell infiltration and expression levels of key genes in LUSC in the Timer database. (**c**) Correlation between immune cell infiltration levels and copy number variation of key genes in NSCLC in the Timer database. (**d**) The relationship between survival time and immune cell infiltration levels in NSCLC patients in the Timer database. *P < 0.05; **P < 0.01; and ***P < 0.001.

The results from the TIMER database demonstrated that in both LUAD and LUSC, the expression of SELE, ICAM1, and ALOX5 positively correlated with the infiltration of the six immune cells mentioned in “Materials and Methods” (Figs. **9a** and **9b**). Meanwhile, the expression of PPARG, MMP9, MMP1, MMP3, and IL6 was only associated with the infiltration of certain immune cells. Specifically, there was a notable negative correlation between PPARG and neutrophils, as well as between MMP3 and B cells exclusively in LUSC. Additionally, a significant negative correlation was observed between MMP1 and B cells in both tissues. Collectively, these findings suggest a close relationship between the expression of these eight key genes and the infiltration of immune cells. Furthermore, the results also reveal a significant correlation between the expression of these eight genes and neutrophil infiltration (*P* < 0.001), irrespective of the NSCLC subtype.

Based on these findings, this study further analyzed the relationship between copy number variations of these eight key genes and immune cell infiltration levels in NSCLC (Fig. **9c**). In LUAD, variations in the somatic copy numbers of IL6 and SELE were significantly associated with the infiltration of six immune cells in NSCLC, while alterations in other key genes were only notably correlated with the infiltration of certain immune cells. Notably, changes in the copy numbers of these eight genes significantly influenced the infiltration of B cells and CD4+ T cells. In LUSC, variations in the somatic copy numbers of MMP3, MMP1, MMP9, PPARG, ALOX5, and ICAM1 exhibited a significant correlation with the infiltration of six immune cells in NSCLC. Alterations in the somatic copy numbers of IL6 and SELE had a considerable impact on the infiltration of the remaining five immune cells, with the exception of CD8+ T cells, where no significant effect was observed.

Finally, Kaplan-Meier curves were generated for NSCLC patients with varying infiltration levels of six immune cells (Fig. **9d**). The results revealed that in LUAD, the infiltration levels of B cells and dendritic cells were significantly correlated with the survival outcomes of NSCLC patients. In contrast, in LUSC, the infiltration levels of the aforementioned six immune cells had a minimal impact on patient survival (*P* > 0.05). These findings imply that different subtypes of NSCLC may be influenced by the infiltration of distinct immune cells, thereby emphasizing the intricate role of immune cells in determining the prognosis of NSCLC.

Furthermore, this study also explored the relationship between the expression of eight pivotal genes and the immunologic labeling of distinct subsets of infiltrating immune cells in NSCLC. As presented in Tables **S3** and **S4**, the expression levels of IL6, MMP3, MMP9, ALOX5, ICAM1, and SELE exhibited a notable correlation with the majority of characteristic markers for the aforementioned six immune cells, regardless of the NSCLC subtype. Remarkably, SELE expression was found to be significantly linked with the diverse markers enumerated. In contrast, PPARG demonstrated this association exclusively in LUAD.

Taken together, these findings further suggest that *H. cordata* can affect the progression of NSCLC and improve the poor prognosis of patients by regulating immune-related mechanisms. This discovery provides new ideas for the development of new treatment options for NSCLC, especially in cellular immunotherapy.

## 4 DISCUSSION

NSCLC lacks significant clinical features, most patients are already in advanced stages at the time of diagnosis, and the prognosis is generally poor. Thus, a breakthrough is urgently needed on how to improve the prognosis. As a natural herbal medicine, *H. cordata* is widely distributed around the world, and in some regions, it is even consumed as a delicious food. Previous studies have shown that *H. cordata* contains a variety of anti-NSCLC components. However, the systematic roles and related mechanisms of these components in anti-NSCLC have not been reported, and whether they can play a positive role in the homology of medicine and food still needs to be further studied. Therefore, in this study, we integrated various biological databases and used a network pharmacology approach integrating target prediction, tissue localization, PPI interaction analysis, enrichment analysis, and target validation to systematically analyze the possible molecular mechanisms of *H. cordata* for the treatment of NSCLC.

In this study, with the help of network pharmacology, molecular docking, molecular dynamics simulation, and multi-omics analysis, various biological networks were constructed through databases such as TCMSP, GEO, PubChem, Swiss Target Prediction, and so on. We analyzed the interactions of potential therapeutic targets of *H. cordata* against NSCLC and identified five core components (quercetin, kaempferol, 2-undecanone, isoramanone, and dodecanal) and eight key targets (IL6, MMP9, PPARG, ICAM1, SELE, MMP3, MMP1, and ALOX5). By analyzing the “disease-pathways-components-targets” network, it was found that the core components of *H. cordata* were highly correlated with NSCLC targets and important molecular mechanisms. These core components might have an important impact on the pathogenesis and treatment strategies of NSCLC, so they deserve our in-depth study.

A search of previous relevant studies revealed that these core components are associated with the regulation of the tumor microenvironment, inhibition of the inflammatory response, and improvement of oxidative stress status. Quercetin is a flavonol compound with a wide range of biological activities, including antioxidant, anticancer, anti-inflammatory, antidiabetic, and antimicrobial activities [56]. There is also current evidence that quercetin shows great potential in the treatment of lung cancer [57]. Chang *et al*. [58] found that quercetin can inhibit Snail-dependent Akt activation by up-regulating the expression of maspin and Snail-independent ADAM9, thus inhibiting the invasion and migration of NSCLC cells. In addition, autophagy is an important mechanism for cancer cells to adapt to stress and plays an important role in tumor cell proliferation, migration, and tumor microenvironment regulation [59, 60]. AMPK is a key signal regulator of autophagy. Quercetin can induce pro-apoptotic autophagy in NSCLC cell lines by up-regulating the expression of SIRT1, increasing the level of p-AMPK, and activating the SIRT1/AMPK signaling pathway [57]. A common flavonoid aglycone found in many natural plants, kaempferol has a wide range of pharmacological properties, such as cardioprotection, neuroprotection, antioxidant, antibacterial, anti-inflammatory, and antidiabetic actions [61]. Kaempferol can cause apoptosis in NSCLC cell lines, suppress cell proliferation, and obstruct migration and invasion, according to a study by Imran *et al*. [61]. Kaempferol can prevent cancer cell invasion by inhibiting the expression activity of MMP9, according to a study by Li *et al*. [62]. 2-Undecanone is a crucial component isolated from *H. cordata*. It not only significantly inhibits the production of TNF-α, IL-1β, and the expression of TLR4 [63], but it also activates the Nrf2-HO-1/NQO-1 signaling pathway, which reduces DNA damage and inflammation caused by benzo(a)pyrene (B[a]P) stimulation and plays a role in the chemoprevention of B[a]P-induced lung tumorigenesis [64]. Although there is no in-depth report on the anticancer activity and related molecular mechanisms of isoramanone and dodecanal, dodecanal has been confirmed to be a diagnostic marker for lung cancer [65]. It is worth noting that based on the results of our study, it can be found that isoramanone not only shows a high degree of connectivity in the “disease-pathways-components-targets” network but also shows good binding activity in the subsequent molecular docking, which indicates that isoramanone has the potential to be a targeted therapy for NSCLC, which has not been reported in the literature so far and is also a highlight of this study. It means that *H. cordata* contains many effective components with anti-tumor activity, which can be used as a new treatment strategy for NSCLC and provide a reliable basis for further study of its pharmacological mechanism.

Organ-tissue localization of *H. cordata*'s potential therapeutic targets against NSCLC was done in this study. It was discovered that several targets were dispersed throughout various organs and tissues, and corresponding targets also existed in the thymus. Therefore, besides the lung, *H. cordata* can also promote the immune activity of distant immune tissues and organs to fight NSCLC. This is consistent with the immunomodulatory activity of *H. cordata* [8]. Furthermore, it was discovered by GO and KEGG enrichment analysis that *H. cordata* affects NSCLC through a variety of biological processes, including the collagen catabolie process, regulation of inflammatory response, regulation of hormone levels, and positive regulation of cell death, and that *H. cordata* could target multiple signaling pathways related to the occurrence and development of cancer and metabolic regulation to play an anti-NSCLC role. Lipid and atherosclerosis and the TNF signaling pathway are not only significantly enriched but also have many potential therapeutic targets, which are considered to be more important pathways in the mechanisms of *H. cordata* against NSCLC. The fundamental cause of atherosclerotic lesions is the disorder of lipid metabolism, which leads to the accumulation of lipids on the arterial wall and triggers a series of complex pathophysiological changes. Similarly, lipid metabolism also plays an important role in lung cancer cells. In the early stages of NSCLC, changes in phospholipid metabolism have been demonstrated [66]. In the study of Zhang *et al*. [67], it was also found that the proliferation of lung cancer cells could be effectively suppressed and their apoptosis could be promoted by inhibiting the key molecules of the cholesterol synthesis pathway. This provides a new idea for the treatment of lung cancer by regulating lipid metabolism. In addition, TIAM2, a lipid metabolism-related gene, not only enhances the resistance to osimertinib and cell motility in LUAD but also contributes to the polarization of M2-like tumor-associated macrophage (M2-TAM) [68]. The combined use of statins in lung cancer patients can not only reduce the risk of death related to lung cancer but also inhibit the production of related lipoproteins and decrease the concentration of inflammatory cells, thereby exerting a certain anti-lung cancer effect [69]. The TNF signaling pathway, which belongs to the exogenous death receptor signaling pathway, plays a pivotal role in cancer immunity and inflammatory reactions. Specifically, TNF-α is capable of inducing epithelial-mesenchymal transition, which facilitates the metastasis of NSCLC [70]. Additionally, elevated expression levels of TNFR2 in lung cancer patients have been linked to lymphatic infiltration, distant metastasis, advanced clinical staging, and a poorer prognosis [71, 72]. Previous research has further corroborated that the combination of TNF attenuated and synergistic mutants with chemotherapy can significantly improve the survival of advanced NSCLC patients [73]. In addition, molecular docking and molecular dynamics simulations showed that the core components generally had good binding activity with key genes, especially quercetin, which could form hydrogen bonds with multiple active sites of MMP9 and could be better embedded into the protein pocket to form a stable conformation. The molecular dynamics simulation showed that the combination was relatively stable and the overall performance of the system was good, which further proved the reliability of the docking results. At the same time, it also meant that the combination of quercetin and MMP9 played a key role in the anti-NSCLC effect of *H. cordata*. As a crucial member of the MMP family, MMP9 has the capacity to break down numerous extracellular matrix proteins. It has been demonstrated to be closely related to the pathology of cancer, including but not limited to invasion, metastasis, and angiogenesis [74]. Notably, MMP9 is overexpressed in a range of malignancies, such as NSCLC, colorectal cancer, and glioblastoma, facilitating tumor cell metastasis [75-77]. Specifically, Chen *et al*. [78] revealed in their research that silencing MMP9 to inhibit the activity of the TGFβ/SMAD pathway significantly reversed the stimulatory effects of TRIM66 overexpression on NSCLC cell migration and invasion. This, in turn, hindered the malignant progression of NSCLC. Moreover, MMP9's involvement in NSCLC metastasis extended beyond its role in the IL-17-GCN5-SOX4-MMP9 axis. Hwang *et al*. [79] also confirmed that down-regulation of MMP2 and MMP9 expression through the AMPK/ SIRT pathway hindered the migration and invasion of NSCLC cells. Collectively, these findings underscore the therapeutic potential of targeting MMP9 in the fight against NSCLC.

Finally, through bioinformatics methods, this study conducted searches and in-depth analyses in major biological databases, exploring the mRNA expression differences, clinical prognosis, protein expression differences, genetic information changes, and immune infiltration in the tumor microenvironment of key genes. The results showed that the expression of these genes is not only closely related to the occurrence and development of NSCLC but is also significantly associated with the prognosis of NSCLC patients. Using the cBioPortal database, this study observed genetic alterations in NSCLC patient populations, with amplification events commonly occurring in pan-cancer cases and the most common alteration being in SELE. The occurrence and development of cancer are closely related to the accumulation of genetic variations, suggesting a potential oncogenic role for SELE mutations. In addition, the results also showed that the expression levels of these genes were closely related to the infiltration of immune cells in NSCLC, particularly showing a strong correlation with neutrophil infiltration. Interestingly, whether in LUAD or LUSC, the expression of SELE was significantly correlated with the various immunological markers listed, which might be related to SELE's role as a member of the selectin family in mediating leukocyte adhesion to inflammatory sites and initiating inflammatory responses [80]. Taken together, these findings provide important clues and implications for revealing the pathogenesis of NSCLC and developing new drugs for the treatment of NSCLC.

## CONCLUSION

The study preliminarily explores the mechanisms of action of *H. cordata* in the treatment of NSCLC through network pharmacology, molecular docking, molecular dynamics simulation, and multi-omics analysis. It is elucidated that *H. cordata* may regulate pathways such as lipid and atherosclerosis, the TNF signaling pathway, and the IL-17 signaling pathway by acting on key targets such as IL6, MMP9, PPARG, ICAM1, SELE, IMMP3, IMMP1, and ALOX5 *via* its core components: quercetin, kaempferol, 2-undecanone, isoramanone, and dodecanal. Furthermore, *H. cordata* potentially plays a role in enhancing the immune activity of immune cells and facilitating the degradation of extracellular matrix proteins. Thus, it combats NSCLC through multi-component, multi-target, and multi-pathway approaches. Among these, the anti-NSCLC activity of isoramanone is reported for the first time and can be further studied as a new component for the treatment of NSCLC.

Although this study integrates big data mining from the HPA, GEPIA, and TIMER databases to validate the findings of network pharmacology, thereby circumventing errors stemming from small sample sizes and incomplete datasets, there are still certain limitations to consider. Primarily, this investigation relies predominantly on qualitative bioinformatics predictions and does not delve into the realm of serum medicinal chemistry. It does not comprehensively analyze alterations in chemical constituents during the extraction and concentration processes of *H. cordata*, nor does it address metabolic changes of *H. cordata* within the human body or the active components entering the bloodstream. Additionally, the identification of key genes solely through the analysis of topological data from biological networks might overlook other compounds, potentially missing more crucial targeted genes. Consequently, the findings of this study require further validation through animal or cell-based experiments to establish a more robust and reliable scientific foundation for the treatment of NSCLC with *H. cordata*.

Furthermore, because most biological databases are inevitably slow to update, the data are often not comprehensive enough, and the targets obtained are often not accurate enough. Some of the predicted results still need more data and a large number of clinical cases to verify. In light of this, our subsequent research will delve deeper into the underlying mechanisms by which *H. cordata* treats NSCLC, with the ultimate goal of identifying markers or therapeutic targets that can aid in clinical predictions of invasion, metastasis, and prognosis. Through rigorous and continued exploration, we aspire to uncover more effective NSCLC treatments, paving the way for improved patient outcomes and an enhanced quality of life.

## Data Availability

The data and supportive information are available within the article.

## LIST OF ABBREVIATIONS

B[a]P: Benzo(a)pyrene
BP: Biological Processes
CC: Cellular Components
COVID-19: Coronavirus Disease 2019
DEGs: Differentially Expressed Genes
DL: Drug-likeness
GO: Gene Ontology
Hb: Hydrogen Bonds
HR: Hazard Ratio
IHC: Immunohistochemistry
KEGG: Kyoto Encyclopedia of Genes and Genomes
LUAD: Lung Adenocarcinoma
LUSC: Lung Squamous Cell Carcinoma
M2-TAM: M2-like Tumor-associated Macrophage
MD: Molecular Dynamics
MF: Molecular Functions
NSCLC: Non-small Cell Lung Cancer
OB: Oral Bioavailability
OS: Overall Survival
PPI: Protein-protein Interaction
Rg: Radius of Gyration
RMSD: Root Mean Square Deviation
RMSF: Root Mean Square Fluctuation
SASA: Solvent-accessible Surface Area
THIF: 3',4',5,7-Tetrahydroxyisoflavanone
