# Supplementary material for: Unraveling the Core Components and Critical Targets of Houttuynia cordata Thunb. in Treating Non-small Cell Lung Cancer through Network Pharmacology and Multi-omics Analysis
Source: Curr Pharm Des. 2024 Oct 21;31(7):540–58. doi: 10.2174/0113816128330427241017110325 (PMC12079317; doi:10.2174/0113816128330427241017110325)
Supplement: Supplementary file 1 [file CPD-31-7-540_SD1.pdf]

Supplementary Material

Unraveling the Core Components and Critical Targets of *Houttuynia cordata* Thunb. in Treating Non-small Cell Lung Cancer through Network Pharmacology and Multi-omics Analysis

Jinyan Yang[1], Yang Li[2], Yan Zhang[1], Ling Xu[1], Jiahui Wang[3], Feng Xing[1,#,*] and Xinqiang Song[1,4,#,*]

[1]College of Life Science, Xinyang Normal University, Xinyang 464000, China; [2]Department of Ultrasound, Xinyang Central Hospital, Xinyang 464000, China; [3]College of International Education, Xinyang Normal University, Xinyang 464000, China; [4]Medical College, Xinyang Normal University, Xinyang 464000, China

 © 2025 Bentham Science Publishers

Table S1. Basic information of active ingredients of *H. cordata* (with oral bioavailability (OB) ≥ 30% and drug-likeness (DL) ≥ 0.18).

Mol ID	Molecule Name	MW	OB（%）	DL
MOL003851	Isoramanone	348.53	39.97	0.51
MOL000422	Kaempferol	286.25	41.88	0.24
MOL004350	Ruvoside_qt	390.57	36.12	0.76
MOL004355	Spinasterol	412.77	42.98	0.76
MOL000098	Quercetin	302.25	46.43	0.28
MOL004345	1-methyl-2-nonacosyl-4-quinolone	566.07	31.54	0.5
MOL004351	C09747	296.35	37.28	0.25

Table S2. Basic information of active ingredients of *H. cordata* (according to the relevant researches).

Mol ID	Molecule Name	MW	OB (%)	DL	Ref.
MOL000121	Decanal	156.3	29.81	0.02	[52]
MOL000701	Quercitrin	448.41	4.04	0.74	[52, 53]
MOL004359	Decanoyl acetaldehyde	198.34	36.04	0.04	[18]
MOL000415	Rutin	610.57	3.2	0.68	[19]
MOL004368	Hyperin	464.41	6.94	0.77	[21]
MOL000924	2-Undecanone	170.33	17.66	0.03	[10]
MOL001644	Dodecanal	184.36	21.52	0.03	[54]
MOL004362	Afzelin	432.41	3.83	0.7	[52]

Table S3. Correlation analysis between key genes and related gene markers of immune cells in LUAD by the TIMER database.

Description	Markers	IL6		MMP3		MMP1		MMP9		PPARG		ALOX5		ICAM1		SELE	
		Cor.	P	Cor.	P	Cor.	P	Cor.	P	Cor.	P	Cor.	P	Cor.	P	Cor.	P
B Cell	CD19	0.081	7.16E-02	0.11	*	-0.072	1.11E-01	0.273	***	-0.111	*	0.087	5.38E-02	0.018	6.84E-01	0.215	***
	CD79A	0.129	**	0.199	***	0.01	8.16E-01	0.294	***	-0.143	**	0.062	1.72E-01	0.005	9.13E-01	0.218	***
	MS4A1	0.014	7.53E-01	0.005	9.11E-01	-0.122	**	0.153	***	-0.002	9.67E-01	0.166	***	0.114	*	0.28	***
CD4 + T Cell	CD2	0.046	3.07E-01	0.126	**	-0.05	2.69E-01	0.29	***	0.117	**	0.371	***	0.193	***	0.136	**
	CD3D	0.071	1.14E-01	0.168	***	0.012	7.85E-01	0.304	***	0.095	*	0.262	***	0.121	**	0.1	*
	CD3E	0.06	1.81E-01	0.099	*	-0.065	1.52E-01	0.317	***	0.072	1.11E-01	0.33	***	0.165	***	0.15	***
CD8 + T Cell	CD8A	0.176	***	0.126	5.02E-02	0.021	6.34E-01	0.235	***	0.127	**	0.188	***	0.113	*	0.131	**
	CD8B	0.154	***	0.126	**	0.012	7.84E-01	0.194	***	0.075	9.82E-02	0.139	**	0.026	5.70E-01	0.113	*
	GZMA	0.197	***	0.13	**	0.04	3.72E-01	0.247	***	0.073	1.04E-01	0.148	***	0.022	6.26E-01	0.092	*
Macrophage	CD68	0.189	***	0.087	5.25E-02	0.083	6.60E-02	0.318	***	0.436	***	0.649	***	0.187	***	0.126	**
Neutrophils	CCR7	-0.025	5.83E-01	0.046	3.03E-01	-0.16	***	0.269	***	0.059	1.93E-01	0.356	***	0.241	***	0.252	***
	ITGAM	0.151	***	0.178	***	0.058	1.98E-01	0.399	***	0.346	***	0.774	***	0.423	***	0.159	***
	SIGLEC5	0.261	***	0.117	**	0.027	5.44E-01	0.332	***	0.301	***	0.637	***	0.311	***	0.253	***
Dendritic Cell	CD1C	-0.156	***	-0.042	3.57E-01	0.133	**	-0.037	4.17E-01	0.128	**	0.514	***	0.4	***	0.123	**
	HLA-DPA1	-0.067	1.40E-01	0.066	1.43E-01	-0.137	**	0.142	**	0.229	***	0.615	***	0.401	***	0.137	**
	HLA-DPB1	-0.088	*	0.026	5.72E-01	-0.158	***	0.139	**	0.209	***	0.631	***	0.38	***	0.133	**
	HLA-DQB1	-0.11	*	0.032	4.75E-01	-0.134	**	0.153	***	0.106	*	0.468	***	0.364	***	0.095	*
	HLA-DRA	-0.029	5.19E-01	0.105	*	-0.093	*	0.144	**	0.248	***	0.605	***	0.391	***	0.129	**
	ITGAX	0.217	***	0.061	1.79E-01	-0.005	9.15E-01	0.443	***	0.251	***	0.658	***	0.329	***	0.169	***
	NRP1	0.203	***	0.16	***	0.023	6.12E-01	0.093	*	0.111	*	0.25	***	0.413	***	0.119	**

*$P < 0.05$; **$P < 0.01$; and ***$P < 0.001$.

Table S4. Correlation analysis between key genes and related gene markers of immune cells in LUSC by the TIMER database.

Description	Markers	IL6		MMP3		MMP1		MMP9		PPARG		ALOX5		ICAM1		SELE	
		Cor.	P	Cor.	P	Cor.	P	Cor.	P	Cor.	P	Cor.	P	Cor.	P	Cor.	P
B Cell	CD19	0.096	*	-0.107	*	-0.152	***	0.205	***	0.081	7.68E-02	0.336	***	0.124	**	0.292	***
	CD79A	0.085	6.50E-02	-0.003	9.51E-01	-0.055	2.27E-01	0.242	***	0.056	2.21E-01	0.335	***	0.133	**	0.286	***
	MS4A1	0.128	**	-0.223	***	-0.222	***	0.134	**	0.173	***	0.357	***	0.131	**	0.399	***
CD4 + T Cell	CD2	0.08	7.95E-02	-0.096	*	-0.04	3.80E-01	0.23	***	0.006	8.93E-01	0.558	***	0.381	***	0.323	***
	CD3D	0.086	6.01E-02	-0.096	*	-0.041	3.70E-01	0.199	***	-0.019	6.76E-01	0.485	***	0.345	***	0.3	***
	CD3E	0.103	*	-0.071	1.23E-01	-0.041	3.77E-01	0.254	***	0	9.98E-01	0.578	***	0.413	***	0.36	***
CD8 + T Cell	CD8A	0.102	*	-0.118	**	-0.056	2.25E-01	0.121	**	-0.004	9.30E-01	0.485	***	0.338	***	0.277	***
	CD8B	-0.011	8.06E-01	-0.203	***	-0.091	*	0.108	*	-0.05	2.73E-01	0.421	***	0.255	***	0.231	***
	GZMA	0.114	*	-0.024	6.05E-01	0.006	8.89E-01	0.059	1.99E-01	-0.023	6.20E-01	0.358	***	0.297	***	0.159	***
Macrophage	CD68	0.201	***	0.08	8.00E-02	0.116	*	0.325	***	0.115	*	0.535	***	0.433	***	0.191	***
Neutrophils	CCR7	0.117	*	-0.15	**	-0.112	*	0.277	***	0.05	2.73E-01	0.558	***	0.353	***	0.388	***
	ITGAM	0.093	*	0.031	5.02E-01	0.055	2.28E-01	0.494	***	-0.014	7.57E-01	0.695	***	0.561	***	0.326	***
	SIGLEC5	0.174	***	-0.015	7.45E-01	0.105	*	0.339	***	0.045	3.28E-01	0.679	***	0.486	***	0.351	***
Dendritic Cell	CD1C	0.028	5.49E-01	-0.173	***	-0.074	1.05E-01	0.163	***	0.127	**	0.45	***	0.184	***	0.337	***
	HLA-DPA1	0.131	**	-0.111	*	-0.005	9.09E-01	0.267	***	0.043	3.53E-01	0.735	***	0.51	***	0.335	***
	HLA-DPB1	0.111	*	-0.121	**	-0.019	6.84E-01	0.311	***	0.052	2.58E-01	0.743	***	0.531	***	0.346	***
	HLA-DQB1	0.102	*	-0.063	1.67E-01	0.018	6.95E-01	0.251	***	0.032	4.91E-01	0.562	***	0.385	***	0.205	***
	HLA-DRA	0.121	**	-0.087	5.86E-02	0.034	4.64E-01	0.253	***	0.015	7.37E-01	0.689	***	0.509	***	0.314	***
	ITGAX	0.128	**	-0.051	2.68E-01	0.007	8.83E-01	0.448	***	0.067	1.46E-01	0.698	***	0.431	***	0.295	***
	NRP1	0.156	***	0.171	***	0.237	***	0.291	***	-0.026	5.66E-01	0.449	***	0.423	***	0.178	***

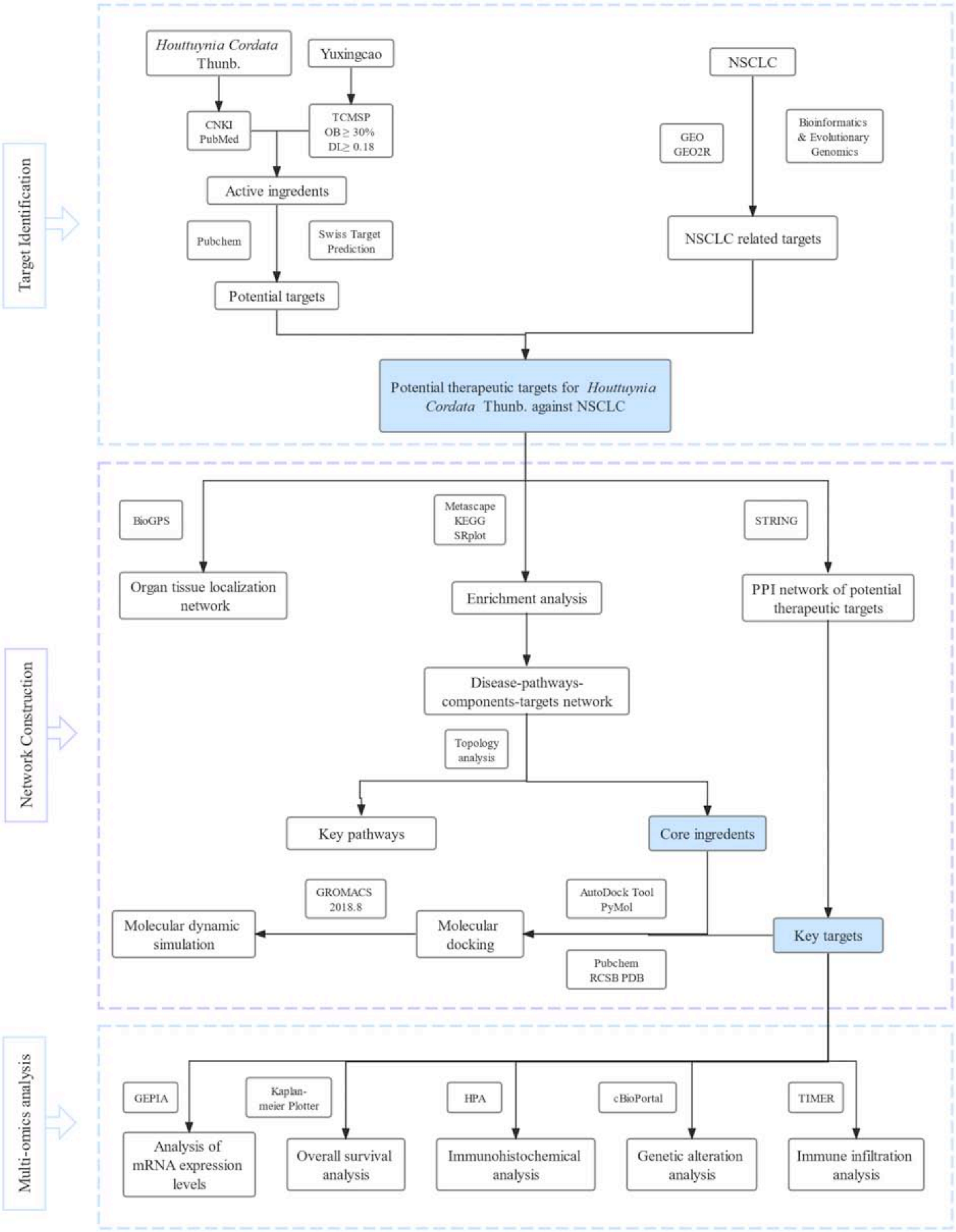

Fig. (S1). The specific experimental process of the study.

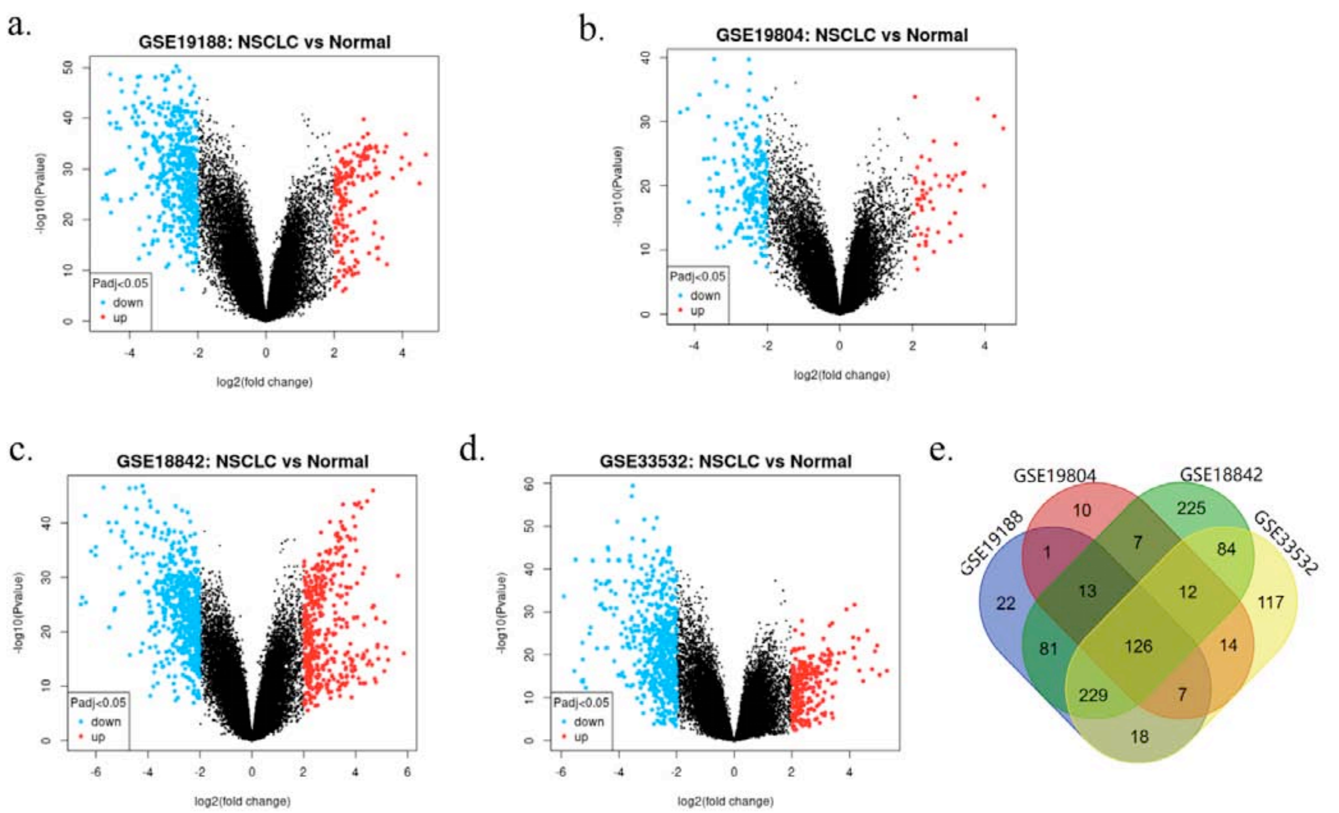

Fig. (S2). Differentially expressed genes in NSCLC. (a-d) Volcano plots of differentially expressed genes in NSCLC from GSE19188(a), GSE19804(b), GSE18842(c) and GSE33532(d) chips. The X and Y-axis represent the - log10 (Pvalue) and log2 (foldchange) of mean normalized values, respectively. Blue dots represent down-regulated genes, red dots represent up-regulated genes, and black dots represent genes that did not meet the screening conditions. (e) Venn diagram of differentially expressed genes in NSCLC.

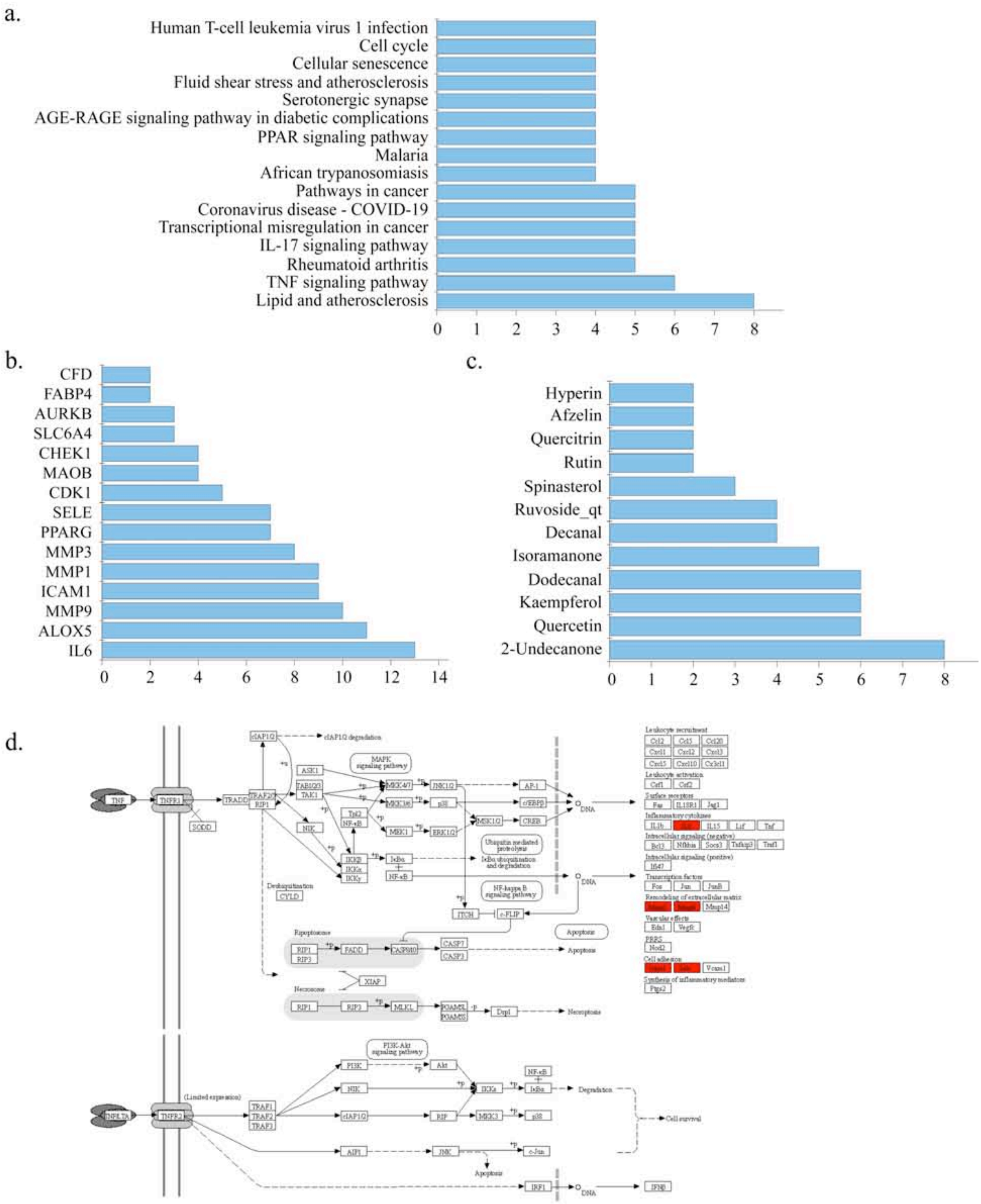
a.
Human T-cell leukemia virus 1 infection
Cell cycle
Cellular senescence
Fluid shear stress and atherosclerosis
Serotonergic synapse
AGE-RAGE signaling pathway in diabetic complications
PPAR signaling pathway
Malaria
African trypanosomiasis
Pathways in cancer
Coronavirus disease - COVID-19
Transcriptional misregulation in cancer
IL-17 signaling pathway
Rheumatoid arthritis
TNF signaling pathway
Lipid and atherosclerosis
0 1 2 3 4 5 6 7 8
b.
CFD
FABP4
AURKB
SLC6A4
CHEK1
MAOB
CDK1
SELE
PPARG
MMP3
MMP1
ICAM1
MMP9
ALOX5
IL6
0 2 4 6 8 10 12 14
c.
Hyperin
Afzelin
Quercitrin
Rutin
Spinasterol
Ruvoside_qt
Decanal
Isoramanone
Dodecanal
Kaempferol
Quercetin
2-Undecanone
0 1 2 3 4 5 6 7 8
d.

e.

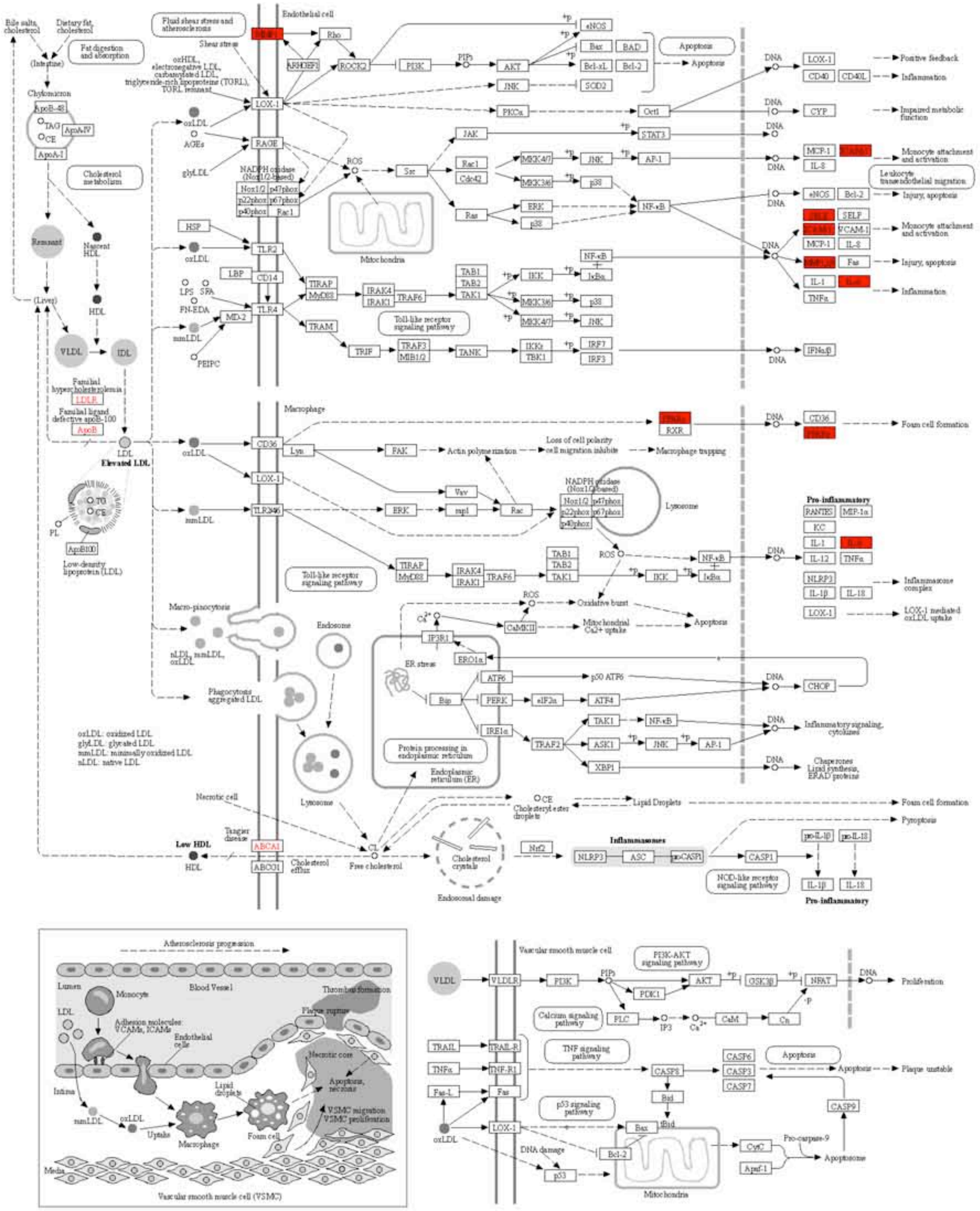

Fig. (S3). (**a-c**) "Degree" values of the pathways, potential therapeutic targets, and compounds. (**d**) Distribution of potential therapeutic targets in the TNF signaling pathway. (**e**) Distribution of potential therapeutic targets in lipid and atherosclerosis. The putative targets implicated in the pathways are shown in red.

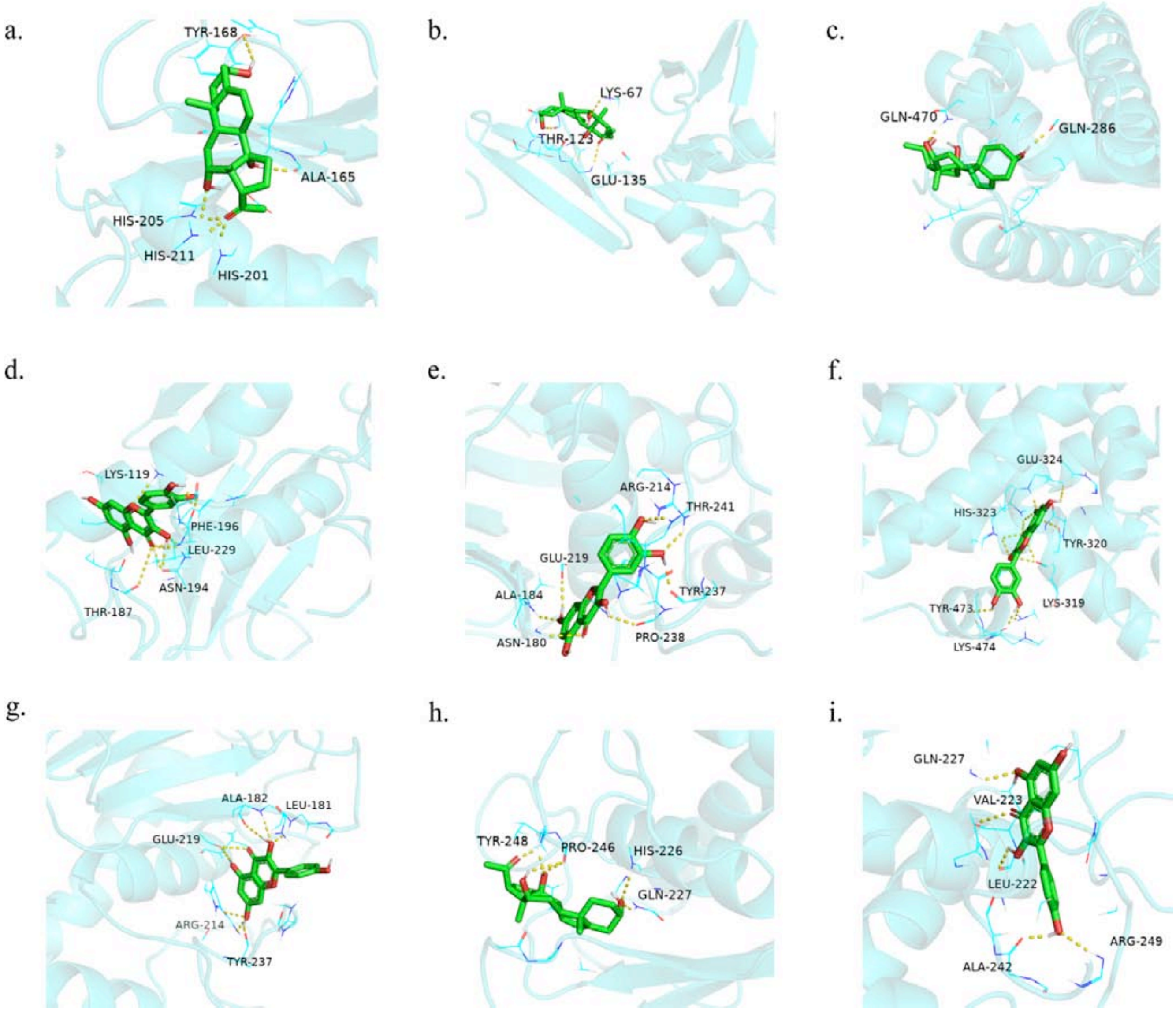

Fig. (S4). Visualization of partial docking results. (**a**) MMP9- Quercetin. (**b**) MMP3-Isoramanone. (**c**) SELE- Isoramanone. (**d**) PPARG- Isoramanone. (**e**) MMP3- Quercetin. (**f**) MMP1- Quercetin. (**g**) PPARG- Quercetin. (**h**) MMP1- Kaempferol. (**i**) MMP9- Isoramanone.
